# Ability of functional performance assessments to discriminate athletes with and without chronic ankle instability : a case-control study

**DOI:** 10.7717/peerj.13390

**Published:** 2022-05-27

**Authors:** Madhura S. Jamsandekar, Vivek Dineshbhai Patel, Ashish J. Prabhakar, Charu Eapen, Justin W.L. Keogh

**Affiliations:** 1 Department of Physiotherapy, Kasturba Medical College, Mangalore, Manipal Academy of Higher Education, Manipal, India; 2 Sports Performance Research Centre, Auckland University of Technology, Auckland, New Zealand; 3 Cluster for Health Improvement, Faculty of Science, Health, Education and Engineering, University of the Sunshine Coast, Queensland, Australia; 4 Faculty of Health Sciences and Medicine, Bond University, Gold Coast, Australia

**Keywords:** Ankle joint (MeSH), Ankle injuries (MeSH), Joint instability (MeSH), Physical functional performance (MeSH)

## Abstract

**Background:**

The decline in motor function associated with chronic ankle instability (CAI) can be assessed using Functional Performance tests. Ankle muscular strength, endurance and range of motion (ROM) has been assessed in previous studies but functional activities such as sprinting and change of direction are less well studied in athletes with CAI. Hence the aim of this study was to determine how sprint, change of direction, ankle isometric strength, endurance and ROM measures may be associated with discriminate athletes with and without CAI.

**Methods:**

One hundred and six participants (CAI: *n* = 53 or no CAI: *n* = 53) provided informed consent to participate in this study. Participants performed three functional performance tests, (30-m sprint test, Modified Illinois change of direction test (MICODT)) and change of direction test. Range of motion for dorsiflexion was measured using weight bearing lunge test and inversion, eversion and plantarflexion using Saunders® digital inclinometer. Strength was assessed using Baseline® hand-held dynamometer for plantarflexors, dorsiflexors, invertors and evertors. Muscular endurance was assessed by single heel raise test and Modified single heel raise test. Between-group comparisons utilised Student’s t-test and Mann-Whitney U-tests, with a number of unique variable and multivariable binomial logistic regression performed to determine which performance measures may discriminate participants with CAI.

**Results:**

The CAI participants performed significantly worse in the three functional performance tests as well as multiple measures of ankle ROM, isometric strength and muscular endurance (*p* < 0.008). While several measures of ROM (plantarflexion and dorsiflexion), strength (inversion and eversion) and both muscular endurance tests were significantly associated with CAI in the univariable analysis, the strongest association was the functional performance tests, especially MICDOT time (odds ratio (95% CI): 0.06 [0.02–0.17], sensitivity 94.3%, specificity 88.7%). Multivariable regression analyses indicated that performance across the functional performance tests were more strongly associated with CAI than any ankle ROM, muscular strength or endurance test. Further, the inclusion of the best ankle range of motion, strength or muscular endurance tests did not significantly improve upon the association of the MICDOT with CAI.

**Conclusions:**

Chronic ankle instability in athletic populations appears to be highly associated with declines in functional performance and to a somewhat lesser extent, ankle range of motion, strength and muscle endurance measures. This may suggest that optimal rehabilitation for athletes with CAI may require a greater focus on improving sprinting speed and change of direction ability in the mid to latter stages of rehabilitation, with regular assessments of these functional performance tests necessary to guide the progression and overload of this training.

## Introduction

One of the most frequent musculoskeletal injuries among athletes is lateral ankle sprain (LAS), accounting for 25% to 30% of injuries (Fong et al., 2007). Out of the total population who experience initial LAS, 40% develop residual symptoms like pain, ankle instability, loss of function, and repeated ankle “giving way” (Arnold et al., 2009). Such symptoms often manifest as motor control deficits over a while, causing an enduring ankle dysfunction known as chronic ankle instability (CAI) (Herzog et al., 2019) Approximately 23% of the athletic population develop CAI (Tanen et al., 2014).

The decline in motor function associated with CAI can be assessed in numerous ways, most commonly with assessments of ankle function (isometric strength, endurance, and ROM) and self-reported measures of function. The assessment of objective and subjective ankle function is vital to tracking rehabilitation milestones and is commonly assessed in the CAI literature. A recent meta-analysis indicates some, but definitely, not all of these muscular strength assessments are associated with CAI (Khalaj et al., 2020). Further, at the same time, a variety of dynamic stability, ankle ROM, and self-reported and therapist-assessed measures of ankle instability/function have been examined in the literature. Such approaches have typically not resulted in very high levels of sensitivity and specificity for CAI (Doherty et al., 2018; Wikstrom et al., 2012) or Cumberland Ankle Instability Tool (CAIT) scores (Rosen, Ko & Brown, 2016). The relative equivalence of the literature for the use of these types of assessments for identifying CAI may reflect these studies’ relatively small sample sizes (*n* = 48–82), mixed populations (recreationally-active and athletic), and the between-study differences in the potential predictor variables assessed (Doherty et al., 2018; Wikstrom et al., 2012; Rosen, Ko & Brown, 2016). Therefore, a relevant question is what combination of tests may better discriminate CAI in athletic populations, with such a question being highly relevant to optimizing their rehabilitation and return to play outcomes.

Functional performance tests (FPTs), when performed together with ankle muscular strength, endurance, and ROM tests, may provide a more comprehensive assessment of performance deficits than ankle function tests alone (Manske & Reiman, 2013; Docherty et al., 2005). Some common FPTs used in the CAI literature include the star excursion balance test and single-leg hop test (Hertel et al., 2006). Unfortunately, the results of the studies, including these assessments, have been inconsistent, perhaps due to the small sample size, vague definition of the CAI population, and lack of comprehensive assessment to best determine the combination of FPTs and ankle function tests to assess athletes with CAI in a single study (Someeh et al., 2015; Groters et al., 2013; Plante & Wikstrom, 2013).

The reductions in general physical and specific training in athletes with CAI are likely to negatively affect their performance ability in sprinting and change of direction (COD) tasks (Hubbard-Turner & Turner, 2015). In these athletes, CAI may further increase their risk of injuries during running, cutting, and sudden COD. Thus, it could be argued that the most appropriate FPTs for athletes of CAI would need to assess sprinting speed and COD as such data would influence their rehabilitation and return to play outcomes. This is highly important as the initial injury-related deficits in ankle ROM, muscle strength, and endurance may recover quicker than sprinting and COD performance reductions. Unfortunately, such sport-specific FPTs have not been commonly assessed in athletes with CAI. The inclusion of sprinting speed and COD FPTs also presents an advantage to clinicians since they are quick to assess, simple to perform, and more feasible than the lab-based assessments of isokinetic strength or force platform-derived balance measures that are relatively commonly performed in research. Therefore, the primary objective of this case-control study was to determine how FPT (sprint and COD) as well as a more comprehensive assessment of ankle function, namely ROM, strength, and muscular endurance scores, may be associated/differ between athletes with and without CAI.

## Materials and Methods

### Participants

This case-control study included 106 participants (53 with and 53 without CAI) between the ages of 18–30 years. The sample size for the study was calculated using G*power software for a multiple linear regression test. For a medium effect size and 13 predictor variables, considering a 1 percent margin of error and 90% study’s statistical power, the total sample size required for this study is 99 (50 per group). The sample size was achieved, whereby we had 53 participants in each group (a total of 106 participants). Using a convenience sampling approach, participants were recruited and 53 matched participants each were divided into two groups between December 2019 and February 2021. These participants were involved in university-level sports, including football (34%), basketball (17.9%), track and field (17.9%), badminton (17%), and volleyball (13.2%). The study was approved by the institutional ethics committee (IEC), KMC Mangalore (KMC MLR 11-19/584). Potential participants read and provided their written informed consent before participation. The study was conducted following the Strengthening the Reporting of Observational Studies in Epidemiology (STROBE) statement.

Participants were divided into two groups, those with and without CAI, based on several criteria. Based on the inclusion criteria stated by International Ankle Consortium (Gribble et al., 2014), athletes were included in the CAI group if they had: (1) History of at least one acute ankle sprain that resulted in inflammation and impaired physical activity. Initial ankle sprain, which occurred ≥12 months prior to testing. (2) The most recent sprain should be ≥3 months. (3) ≥2 episodes of “giving way” and/or recurrent ankle sprain and/or feelings of instability at the ankle 6 months prior to the study enrolment that did not result in an ankle sprain. (4) a score of ≤24 on the CAIT scale. For a participant who reported a history of a bilateral ankle injury, the limb with the maximum number of give way episodes and the lowest CAIT score was considered. The participants without a history of LAS or ankle instability on both sides and who did not have self-reported functional loss were placed in the non-CAI group. Exclusion criteria consisted of Individuals with a history of previous surgeries of lower extremity musculoskeletal structures, including bone, ligaments, and/or nerve injury that could affect their performance in functional performance test; or any acute injury to musculoskeletal structures of the lower limb, either sprain, strain, or fracture within 3 months prior to testing.

### Procedure

Participants performed 10 min of a self-selected warm-up prior to performing the assessments. Assessments of ankle ROM were performed first, followed by muscular strength, muscular endurance tests and finally, FPTs. Participants were provided with a demonstration and verbal instructions prior to each test, with familiarization and two submaximal trials also provided for the FPTs, *i.e*., 30-m sprint test, MICODT, and change of direction test. All the FPTs were performed in random order. Each test was performed three times, and the best time of the three trials was recorded. The participant was given a rest interval of 30 s after each trial and 1 min after three trials of each FPT.

### Assessment of FPTs

**Figure 1:**
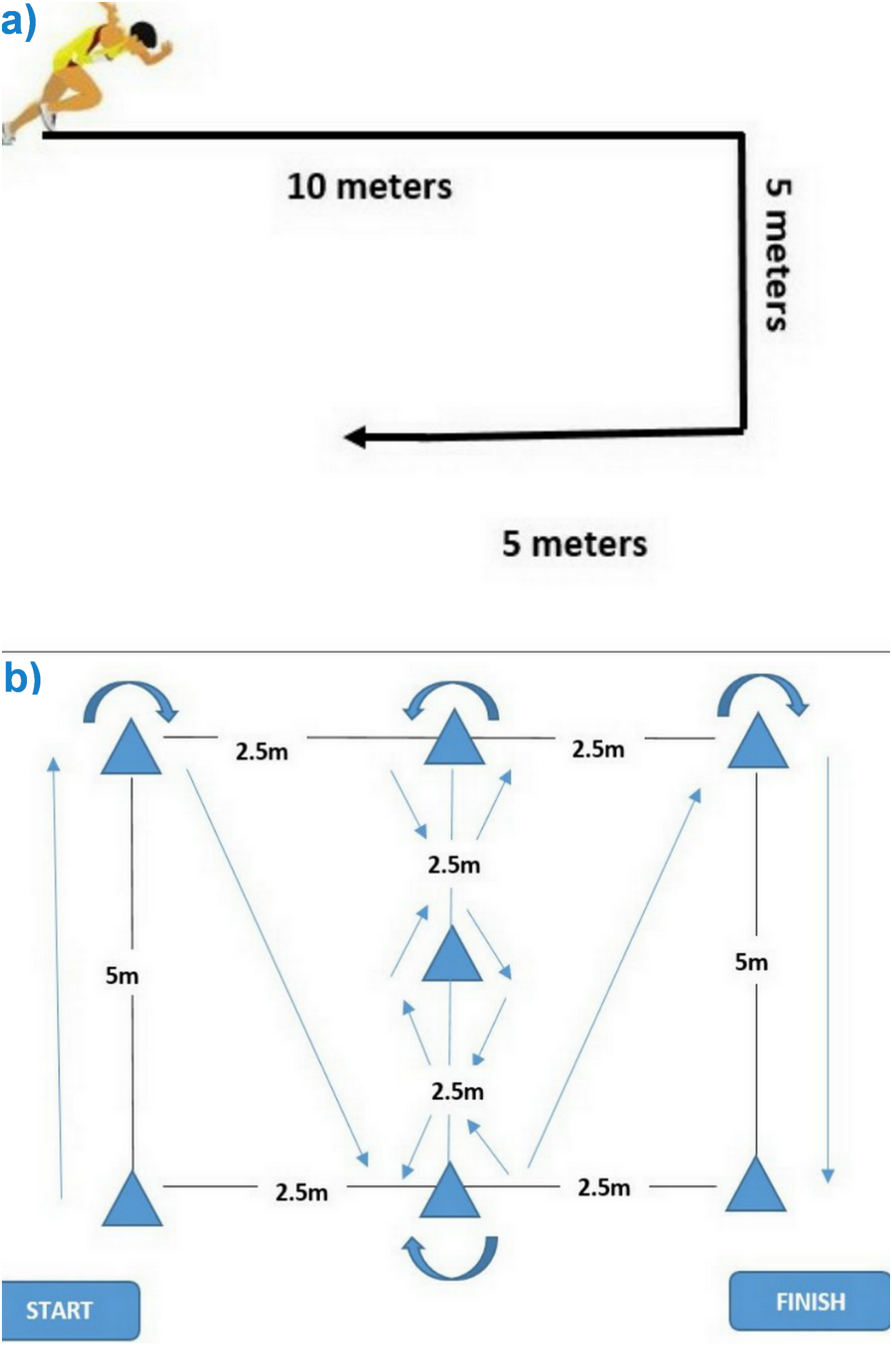
Schematic representations of the change of direction (COD) (A) and modified Illinois change of direction test (MICODT) (B) tests.

The three FPTs were performed on a natural grass terrain, and participants wore running shoes without spikes. In the 30-m sprint test, the participant starts from a stand start and, on the researcher’s command, sprints for 30 m to the finish line, with timing assessed by trained researchers using a stopwatch. This sprinting speed assessment by a stopwatch has been reported to have excellent reliability (ICC = 0.95–0.97) (Hetzler et al., 2008), a result consistent with our own laboratory (ICC = 0.93). The COD test was adapted and modified from a study conducted by Grazioli et al. (2020) by adding a sharp 90 degrees turn for assessing COD and is described pictorially in Fig. 1A. We found excellent reliability (ICC = 0.91) for this modified COD test. The MICODT is an adaption of the Illinois Agility Test that is conducted over a shorter distance better to replicate change of direction requirements in ball sports (see Fig. 1B). The MICDOT was also reported to have excellent relative reliability ICC = 0.99 (Hachana et al., 2014).

### Assessment of ROM

Range of motion was assessed in a random order for ankle dorsiflexion (DF), plantarflexion (PF), inversion (INV), and eversion (EVE) using a Saunders® digital Inclinometer (The Saunders Group, Inc. Chaska, MN, USA). Ankle DF was assessed using Weight Bearing Lunge Test (WBLT), where the participants performed the test with a digital inclinometer placed 15 cm below the tibial tuberosity (Hall & Docherty, 2017). The WBLT has proven to be a reliable tool for detecting ROM deficits in CAI subjects, with its intra-rater reliability being ICC = 0.89–0.98 (Hall & Docherty, 2017). ROM for the PF was assessed in a supine position with the lower limb resting on the table with the knee extended so that the foot and ankle were off the table, while ROM for INV and EVE were assessed in a hook lying position according to previously described methods with its intra-rater reliability being ICC = 0.81–0.96 (Fraser et al., 2017).

### Assessment of muscle strength

Isometric muscle strength was assessed randomly for ankle plantarflexors, dorsiflexors, invertors, and evertors using a Baseline® digital push-pull dynamometer (Fabrication Enterprises Inc., Elmsford, NY, USA). Isometric strength for ankle dorsiflexors, invertors, and evertors was assessed in the supine position and plantarflexors in the prone position, with force applied using a dynamometer at the metatarsal heads. Each participant was instructed to perform three maximal voluntary isometric contractions for each muscle group, each held for 3–5 s. All the assessments were performed supine with the participant’s hips and knees extended and the lower limb stabilized proximal to the ankle joint. These assessments have been reported to have excellent inter-rater reliability (ICC = 0.77 to 0.88) (Spink, Fotoohabadi & Menz, 2010). The average of three trials for the above tests were used for analysis. The measures were not normalized by body mass.

### Assessment of muscle endurance tests

The single heel raise test (SHRT) and modified single heel raise test (MSHRT) were used to assess muscular endurance (Park et al., 2019). For the SHRT, the participants stood on the leg that was being evaluated, with the other leg raised off the ground and its knee flexed to approximately 90 degrees. They performed single-leg heel raises at a cadence of one per second, with the number of repetitions recorded (Lunsford & Perry, 1995). This test was reported to have excellent reliability ICC = 1.00 (Hébert-Losier et al., 2017). The MSHRT was performed with the participants standing on their affected leg, whereby they were required to rise onto the ball of their feet and hold this position for maximum time. When the heel touched the floor, the test was terminated, and time was recorded in seconds (Park et al., 2019). All the assessments were performed in a single session, and it took approximately 25 min for the complete assessment.

### Statistical Analyses

Shapiro-Wilk tests identified normal distribution for all three FPTs and non-normal distribution for most of the ankle ROM, strength, and muscular endurance variables. Thus, between-group comparisons were conducted using Student’s t-tests for the three FPTs while the Mann-Whitney U-test for the remaining outcomes. Cohen’s D for the between group differences was determined by calculating the mean difference between two groups, and dividing it by the pooled standard deviation using Microsoft Excel.

Univariable and multivariable binomial logistic regression were used to gain insight into the odds of the participants having CAI based on the FPT and ankle function tests performed in this study. Specifically, univariable binomial linear regressions were performed on each of the FPT, ROM, isometric strength, and muscular endurance measures (Machin et al., 2018; Pearce, 2016). The first series of multivariable binomial regression analyses, which resulted in four separate regression models, were separately conducted on each of the four categories (FPT, ROM, isometric strength, and muscular endurance) of assessments in order to determine whether either of these four categories were more highly associated with CAI. Finally, a multivariable binomial regression analysis was performed using the best predictor from each of the four categories of assessments to determine which combination resulted in the highest association with CAI. Odds ratios with a 95% confidence interval (95% CI) were calculated for all predictor variables. Their sensitivity and specificity were also calculated for all binomial regression models, with a cut-off value of 0.5. For all statistical comparisons, *p* < 0.05 was considered statistically significant.

## Results

Table 1 summarizes the CAI and non-CAI participants’ demographic characteristics and functional outcomes, *i.e*., FPT, ROM, isometric strength, and muscular endurance outcomes. No significant differences were in sex, age, height, body mass, and body mass index (BMI). A significantly reduced CAIT score was observed for CAI compared to non-CAI participants.

**Table 1:** Demographic characteristics of the CAI and non-CAI participants.

Outcomes	CAI participants (*n* = 53)	Non-CAI participants (*n* = 53)	*p* value
Age (years)	21.9 ± 2.6	21. ± 2.1	0.306
Sex (Male/Female)	33/20	37/16	0.416
Height (cm)	171.6 ± 7.9	172.1 ± 9.4	0.588
Body mass (kg)	64.5 ± 10.1	64.7 ± 11.3	0.939
BMI (kg/m^2^)	22.0 ± 2.7	21.5 ± 3.0	0.310
CAIT	19.5 ± 2.8	30.0 ± 0.0	<0.001*

**Notes:**

* Statistically significant (*p* < 0.05).

All data is presented as Mean ± SD, except for sex which is presented as Males/Females.

CAI, Chronic ankle instability; *n*, number of participants; cm, centimetres; kg, kilograms; m, meter; CAIT, Cumberland Ankle Instability Tool.

A summary of the FPT, ROM isometric muscle strength, and endurance outcomes for both groups is provided in Table 2. Student’s t-test revealed significant differences between the two groups for the primary outcomes of speed, MICOD, and COD, whereby participants with CAI were slower than the healthy participants. Significant between-group differences favoring the non-CAI group were also observed for ROM (plantarflexion and dorsiflexion), isometric strength (ankle invertor and evertor), and muscular endurance tests (SHRT and MSHRT).

**Table 2:** Between-group comparison of Demographic characteristics, functional performance and ankle tests for CAI and healthy participants.

Outcome	CAI group (*n* = 53)	Non-CAI group (*n* = 53)	*p* value	Cohen’s D (95% CI)
FPT				
30 m sprint (s)	5.67 ± 0.58	4.97 ± 0.63	<0.001*	1.16 [0.72–1.60]
MICODT (s)	14.52 ± 0.78	12.57 ± 0.87	<0.001*	2.38 [1.78–2.987]
COD (s)	5.81 ± 0.45	5.14 ± 0.51	<0.001*	1.39 [0.92–1.85]
Ankle ROM				
PF (°)	39.0 (37.0–40.0)	40.6 (38.3–44.0)	0.008*	−0.53 [−0.92 to −0.14]
DF (°)	34.3 (33.0–36.3)	39.0 (37.0–42.0)	<0.001*	−1.38 [−1.80 to −0.95]
INV (°)	36.6 (35.3–38.3)	37.0 (36.0–38.0)	0.951	0.01 [−0.37 to 0.39]
EVE (°)	17.3 (15.3–18.3)	17.6 (16.6–19.0)	0.209	−0.25 [−0.63 to 0.14]
Strength				
PF (kg)	12.3 (10.6–14.0)	13.3 (11.0–14.6)	0.317	−0.20 [−0.58 to 0.19]
DF (kg)	10.6 (8.3–12.0)	12.0 (8.0–13.6)	0.266	−0.22 [-0.60 to 0.17]
INV (kg)	6.0 (5.0–7.0)	7.6 (6.6–8.3)	<0.001*	−0.82 [−1.26 to −0.40]
EVE (kg)	5.3 (4.6–6.0)	8.0 (7.0–9.0)	<0.001*	−1.67 [−2.16 to −1.17]
Muscular endurance				
SHRT (reps)	30.0 (25.0–35.0)	39.0 (35.0–41.0)	<0.001*	−0.84 [−1.25 to 0.43]
MSHRT (s)	34.0 (25.0–45.0)	55.0 (39.6–64.0)	<0.001*	−0.64 [−1.04 to −0.23]

**Notes:**

* Statistically significant (*p* < 0.05).

All functional performance data is presented as Mean ± SD except for ankle ROM, isometric strength and muscular endurance tests are expressed as medians (interquartile ranges).

CAI, Chronic ankle instability; *n*, number of participants; CI, confidence interval; FPT, Functional performance test; m, meter; s, seconds; MICODT, Modified Illinois Change of Direction Test; COD, Change of Direction test; ROM, range of motion; PF, plantarflexion; kg, kilograms; DF, dorsiflexion; INV, inversion; EVE, eversion; SHRT, Single heel raise test; reps, repetitions; MSHRT, Modified heel raise test.

Table 3 provides a summary of the univariate binomial logistic regressions which looked to determine the association between CAI and all of the individual tests in FPT, ROM, isometric strength, or muscular endurance categories. Significant predictors of having CAI included measures of functional performance (MICODT), ROM (plantarflexion and dorsiflexion), isometric strength (invertor and evertor) and muscular endurance (SHRT and MSHRT).

**Table 3:** Summary of univariable binomial logistic regressions for determining association with CAI.

Predictor	Estimate	SE	*p* value	Odds ratio (95% CI)	Specificity (%)	Sensitivity (%)
FPT						
30 m sprint	−2.100	0.469	<0.001*	0.12 [0.05–0.31]	77.4	73.6
MICODT	−2.830	0.548	<0.001*	0.06 [0.02–0.17]	94.3	88.7
COD	−3.180	0.657	<0.001*	0.04 [0.01–0.15]	83.0	73.6
ROM						
PF	0.133	0.052	0.011*	1.14 [1.03–1.27]	64.2	56.6
DF	0.492	0.102	<0.001*	1.64 [1.34–2.00]	77.4	71.7
INV	−0.005	0.084	0.950	1.00 [0.84–1.17]	37.7	54.7
EVE	0.130	0.103	0.209	1.14 [0.93–1.39]	49.1	54.7
Strength						
PF	0.070	0.070	0.314	1.07 [0.94–1.23]	50.9	60.4
DF	0.080	0.072	0.263	1.08 [0.94–1.25]	54.7	62.3
INV	0.565	0.154	<0.001*	1.76 [1.30–2.38]	69.8	67.9
EVE	1.270	0.246	<0.001*	3.56 [2.20–5.77]	86.8	81.1
Muscular endurance						
SHRT	0.102	0.028	<0.001*	1.11 [1.05–1.17]	67.9	67.0
MSHRT	0.032	0.011	0.002*	1.03 [1.01–1.05]	69.8	62.3

**Notes:**

* Statistically significant (*p* < 0.05).

CAI, Chronic ankle instability; SE, standard error; CI, confidence interval; FPT, Functional performance test; m, meter; MICODT, Modified Illinois Change of Direction Test; COD, Change of Direction test; ROM, range of motion, PF, plantarflexion; DF, dorsiflexion; INV, inversion; EVE, eversion; SHRT, single heel raise test; MSHRT, Modified single heel raise test.

A summary of the multivariable binomial logistic regressions determining the association between CAI and each of the four categories (FPT, ROM, isometric strength, or muscular endurance) is presented in Table 4. The most excellent specificity and sensitivity were observed for the FPTs, with the muscular endurance tests providing the lowest specificity and sensitivity.

**Table 4:** Summary of the multivariable binomial logistic regressions for determining association with CAI based on four categories of variables (*i.e*., FPT, ROM, isometric strength, or muscular endurance).

Predictor	Estimate	SE	*p* value	Odds ratio (95% CI)	Specificity (%)^a^	Sensitivity (%)^a^
FPT					94.3	84.9
30 m sprint	−0.147	−0.665	0.825	0.86 [0.23–3.18]		
MICODT	−2.555	0.603	<0.001*	0.08 [0.02–0.25]		
COD	−1.288	0.811	0.112	0.28 [0.06–1.35]		
ROM					79.2	79.2
PF	0.170	0.073	0.020*	1.19 [1.03–1.37]		
DF	0.489	0.103	<0.001*	1.63 [1.33–2.00]		
INV	−0.051	0.106	0.631	0.95 [0.77–1.17]		
EVE	0.021	0.137	0.877	1.02 [0.78–1.34]		
Strength					88.7	81.1
PF	−0.058	0.122	0.635	0.94 [0.74–1.20]		
DF	−0.166	0.130	0.204	0.85 [0.66–1.09]		
INV	−0.294	0.285	0.302	0.75 [0.43–1.30]		
EVE	1.652	0.356	<0.001*	5.22 [2.60–10.49]		
Muscular endurance					73.6	67.9
SHRT	0.085	0.029	0.003*	1.09 [1.03–1.15]		
MSHRT	0.019	0.011	0.103	1.02 [1.00–1.04]		

**Notes:**

* Statistically significant (*p* < 0.05).

CAI, Chronic ankle instability; FPT, Functional performance test; ROM, range of motion; SE, standard error; CI, confidence interval; m, meter; MICODT, Modified Illinois Change of Direction Test; COD, Change of Direction test; PF, plantarflexion; DF, dorsiflexion; INV, inversion; EVE, eversion; SHRT, single heel raise test; MSHRT, Modified single heel raise test.

a Specificity and sensitivity values are provided for each of the four binomial linear regressions on the same line of the table in which the title of the category of assessments is provided.

Table 5 summarizes the multivariable binomial logistic regression, which included the strongest predictor from each of the FPT, ROM, isometric strength, and muscular endurance categories. The MICDOT and evertor strength remained the only significantly associated outcomes, with very high specificity and sensitivity observed for this model.

**Table 5:** Summary of the multivariable binomial logistic regressions for determining association with CAI based on the most highly associated variable from each of the four categories of assessments (*i.e*., FPT, ROM, isometric strength, or muscular endurance).

Predictor	Estimate	SE	*p* value	Odds ratio (95% CI)	Specificity (%)^a^	Sensitivity (%)^a^
MICODT	−2.143	0.523	< 0.001*	0.18 [0.04–0.33]		
DF ROM	0.157	0.187	0.401	1.17 [0.81–1.69]		
EVE Str	0.656	0.317	0.038*	1.93 [1.04–3.58]		
SHRT	0.005	0.047	0.107	1.01 [0.92–1.20]	94.3	92.5

**Notes:**

* Statistically significant (*p* < 0.05).

CAI, Chronic ankle instability; FPT, Functional performance test; ROM, range of motion; SE, standard error; CI, confidence interval; MICODT, Modified Illinois Change of Direction Test; DF, dorsiflexion; EVE Str, eversion strength; SHRT, single heel raise test.

a The specificity and sensitivity values provided on the last line of the table are those for the entire model.

## Discussion

One of the novel results of the current study was that significant functional performance deficits, as measured in the 30-m sprint, MICODT, and COD tests were identified in athletes with CAI compared to those without CAI. The question then emerges about how such functional performance deficits may be reduced in athletes with CAI. One approach might be to determine how characteristics such as functional performance in sprinting and change of direction task and more ankle joint specific tests of ROM, strength, and muscular endurance may be associated with CAI. Identifying such characteristics may then inform the primary focus of rehabilitation programs and return to play guidelines for athletes with CAI.

A series of univariable and multivariable binomial logistic regressions were performed to achieve this aim. The univariable binomial logistic regression results indicated that the test most associated with CAI was the MICDOT. The association between the MICDOT and CAI appears more excellent than the results of any other similar studies, including the posterior talar glide test (Doherty et al., 2018) or a variety of measures of dynamic postural stability or self-reported ankle function (Wikstrom et al., 2012). This may suggest that the MICDOT could be helpful to include in the regular assessment batteries for athletes with CAI, with their performance on repeated assessments of this test used as a part of their readiness to return to play decisions in the mid-latter stages of their rehabilitation.

A series of multivariable binomial linear regression analyses were also performed to identify the combination of most highly associated tests with CAI. The first of these regression analyses was performed within each of the four categories of functional and ankle assessments. The FPTs had the strongest associations to CAI, with the lowest associations observed for the muscular endurance tests. The final multivariable binomial linear regression involved the inclusion of the most highly associated variable from each of the four categories of assessments. While this model, which included the MICDOT, dorsiflexion ROM, eversion strength, and single heel raise muscular endurance, was highly associated with CAI, such results were not substantially improved from that of the univariable binomial analysis involving the MICDOT in isolation. Combining the functional performance tests had the highest sensitivity at 84.9% (Table 5) and, therefore, should be an integral part of the physical examination in cases of CAI. The reason could be as these tests mimic the functional activities commonly involved that might lead to ankle instability. The MICODT appears to be the most appropriate test among the functional tests as it has the highest sensitivity. On the other hand, the two endurance tests were found to be the least sensitive, thus indicating that it may not be much of a factor contributing to the ankle instability compared to the strength required to provide stability to the joint. Nevertheless, when the battery of tests is done for ankle instability, including ROM, strength, functional tests, and endurance, the sensitivity increased to 92.5%, thus indicating that MICODT, dorsiflexion ROM, the strength of the evertors, and SHRT tests may provide some additional insight into the factors associated with ankle instability.

It is, however, unlikely that most athletes in the early-mid stages of rehabilitation from CAI would be able to safely perform high-intensity change of direction tasks as required in the MICDOT. In this stage of their rehabilitation, it might be prudent for the athlete to focus on improving aspects of ankle function that are most associated with CAI. Results of the current study indicated that participants with CAI typically had numerous significant reductions in ankle ROM, isometric strength, and muscular endurance compared to those without CAI, with all of these muscle function characteristics potentially influential in determining their functional performance. Specifically, participants with CAI had significantly reduced plantarflexion and dorsiflexion ROM, inversion and eversion strength, and muscular endurance as assessed in two different single leg heel raise tests. These findings are consistent with previous research, whereby deficits in a variety of measures of ankle function have been observed in individuals with CAI (Gribble et al., 2014; Silva et al., 2013; Wisthoff et al., 2019).

Interestingly, in our study, participants with CAI showed significantly reduced plantarflexor performance in the two single leg muscular endurance tests but failed to demonstrate any significant differences in isometric plantarflexor muscular strength. Further, univariable binomial regression analyses indicated that of the four muscular strength and two muscular endurance tests, only plantarflexion and dorsiflexion strength were not significantly associated with CAI. Such results may have considerable clinical relevance to the assessments and exercise prescription required for athletes with CAI.

Concerning the most appropriate ankle joint assessments for athletes with CAI, it is unclear what the relative differences in the predictive ability of the isometric dynamometry of the plantarflexors and dorsiflexors compared to simple, single leg muscular endurance tests represent. While our results suggest muscular endurance may be more critical than isometric strength, it may also be that the greater multiplanar ankle control required in the single-leg muscle endurance tests is more reflective of the challenges imposed on the ankle during high-intensity movements such as change of direction and sprinting (Bicici, Karatas & Baltaci, 2012). Thus, functional single-leg muscular endurance tests that are inexpensive, quick to administer, and require no equipment may be a more appropriate measure to quickly assess the rehabilitative progress in athletes with CAI than isometric dynamometry.

Exercise prescription applications of these results also suggest a focus on increasing ankle eversion (and perhaps inversion) strength, and single-leg calf raise performance should be emphasized. A recent training study involving 54 athletes with a recurrent lateral ankle sprain provides longitudinal support for this view. These athletes completed either 6 weeks of resistance training (involving theraband-resisted inversion, eversion, plantar flexion, dorsiflexion, and heel and forefoot raise) or balance training (primarily using a BOSU ball and often performed on one leg) (Wang, Yu & Kim, 2021). At the end of the 6 weeks of training, participants in both groups tended to show significant improvement in ankle strength, dynamic balance, hopping, and self-reported ankle function (Wang, Yu & Kim, 2021). Additional research is still, however, required to demonstrate whether such improvements in function would translate to improved rehabilitative outcomes for athletes with CAI.

One of the limitations of our study was that our case-control design does not allow us to predict the development of CAI; instead, it allows us to gain insight into factors associated with athletes who already have CAI. For studies wishing to determine what factors may predict the development of CAI, a well-designed prospective cohort study would provide insight into the potential predictive power of the FPTs and ankle function tests. Another potential limitation of the study was the potential for some fatigue effects during the testing session. However, pilot testing and participant feedback suggested such fatigue effects were minimal. In addition, we recorded the strength measures in absolute terms rather than normalizing to body mass which may be considered another limitation. The study results may also be limited to individuals with similar characteristics to those of the participants, *i.e*., young adult athletic populations with moderate levels of chronic ankle instability.

## Conclusions

Athletes with CAI demonstrated significantly slower sprinting and COD times, ankle ROM, isometric strength, and muscular endurance, which were indicative of their reduced functional performance compared to non-CAI participants. Logistic regression results indicated that performance in the MICODT was most predictive of CAI, with a specificity of 94.3% and sensitivity of 84.9%. Further, ROM of the plantarflexion and dorsiflexors, eversion isometric strength, and the number of repetitions performed in the SHRT were also predictive of CAI. These results indicate that athletes with CAI may initially need to focus their rehabilitation on improving plantarflexion and dorsiflexion ROM, eversion isometric strength, single leg calf muscular endurance, and focus in the later stages on improving their change of direction ability before they safely return to play.

## Supplemental Information

10.7717/peerj.13390/supp-1 Supplemental Information 1 Raw data of functional performance tests and comprehensive ankle assessment.

10.7717/peerj.13390/supp-2 Supplemental Information 2 Strobe checklist.

## Additional Information and Declarations

### Data Availability

The following information was supplied regarding data availability: The raw data of CAI athletes and those without CAI is available in the Supplemental File.

## References

[ref-1] Arnold BL, Linens SW, de la Motte SJ, Ross SE (2009). Concentric evertor strength differences and functional ankle instability: a meta-analysis. Journal of Athletic Training.

[ref-2] Bicici S, Karatas N, Baltaci G (2012). Effect of athletic taping and kinesiotaping® on measurements of functional performance in basketball players with chronic inversion ankle sprains. International Journal of Sports Physical Therapy.

[ref-3] Docherty CL, Arnold BL, Gansneder BM, Hurwitz S, Gieck J (2005). Functional-performance deficits in volunteers with functional ankle instability. Journal of Athletic Training.

[ref-4] Doherty C, Bleakley C, Hertel J, Caulfield B, Ryan J, Delahunt E (2018). Clinical tests have limited predictive value for chronic ankle instability when conducted in the acute phase of a first-time lateral ankle sprain injury. Archives of Physical Medicine and Rehabilitation.

[ref-5] Fong DT, Hong Y, Chan LK, Yung PS, Chan K (2007). A systematic review on ankle injury and ankle sprain in sports. Sports Medicine.

[ref-6] Fraser JJ, Koldenhoven RM, Saliba SA, Hertel J (2017). Reliability of ankle-foot morphology, mobility, strength, and motor performance measures. International Journal of Sports Physical Therapy.

[ref-7] Grazioli R, Lopez P, Machado CLF, Farinha JB, Fagundes AO, Voser R, Reischak-Oliveira A, Setuain I, Izquierdo M, Pinto RS, Cadore EL (2020). Moderate volume of sprint bouts does not induce muscle damage in well-trained athletes. Journal of Bodywork and Movement Therapies.

[ref-8] Gribble PA, Delahunt E, Bleakley C, Caulfield B, Docherty C, Fourchet F, Fong D, Hertel J, Hiller C, Kaminski T, McKeon P, Refshauge K, van der Wees P, Vicenzino B, Wikstrom E (2014). Selection criteria for patients with chronic ankle instability in controlled research: a position statement of the international ankle consortium. Journal of Athletic Training.

[ref-9] Groters S, Groen BE, van Cingel R, Duysens J (2013). Double-leg stance and dynamic balance in individuals with functional ankle instability. Gait & Posture.

[ref-10] Hachana Y, Chaabène H, Ben Rajeb G, Khlifa R, Aouadi R, Chamari K, Gabbett TJ (2014). Validity and reliability of new agility test among elite and subelite under 14-soccer players. PLOS ONE.

[ref-11] Hall EA, Docherty CL (2017). Validity of clinical outcome measures to evaluate ankle range of motion during the weight-bearing lunge test. Journal of Science and Medicine in Sport.

[ref-12] Hertel J, Braham RA, Hale SA, Olmsted-Kramer LC (2006). Simplifying the star excursion balance test: analyses of subjects with and without chronic ankle instability. Journal of Orthopaedic & Sports Physical Therapy.

[ref-13] Herzog MM, Kerr ZY, Marshall SW, Wikstrom EA (2019). Epidemiology of ankle sprains and chronic ankle instability. Journal of Athletic Training.

[ref-14] Hetzler RK, Stickley CD, Lundquist KM, Kimura IF (2008). Reliability and accuracy of handheld stopwatches compared with electronic timing in measuring sprint performance. Journal of Strength and Conditioning Research.

[ref-15] Hubbard-Turner T, Turner MJ (2015). Physical activity levels in college students with chronic ankle instability. Journal of Athletic Training.

[ref-16] Hébert-Losier K, Wessman C, Alricsson M, Svantesson U (2017). Updated reliability and normative values for the standing heel-rise test in healthy adults. Physiotherapy.

[ref-17] Khalaj N, Vicenzino B, Heales LJ, Smith MD (2020). Is chronic ankle instability associated with impaired muscle strength? Ankle, knee and hip muscle strength in individuals with chronic ankle instability: a systematic review with meta-analysis. British Journal of Sports Medicine.

[ref-18] Lunsford BR, Perry J (1995). The standing heel-rise test for ankle plantar flexion: criterion for normal. Physical Therapy.

[ref-19] Machin D, Campbell MJ, Tan SB, Tan SH (2018). Sample sizes for clinical, laboratory and epidemiology studies, 4th edition. https://www.wiley.com/en-us/Sample+Sizes+for+Clinical%2C+Laboratory+and+Epidemiology+Studies%2C+4th+Edition-p-9781118874943.

[ref-20] Manske R, Reiman M (2013). Functional performance testing for power and return to sports. Sports Health.

[ref-21] Park YH, Park SH, Kim SH, Choi GW, Kim HJ (2019). Relationship between isokinetic muscle strength and functional tests in chronic ankle instability. The Journal of Foot and Ankle Surgery.

[ref-22] Pearce N (2016). Analysis of matched case-control studies. BMJ.

[ref-23] Plante JE, Wikstrom EA (2013). Differences in clinician-oriented outcomes among controls, copers, and chronic ankle instability groups. Physical Therapy in Sport.

[ref-24] Rosen A, Ko J, Brown C (2016). A multivariate assessment of clinical contributions to the severity of perceived dysfunction measured by the cumberland ankle instability tool. International Journal of Sports Medicine.

[ref-25] Silva JR, Magalhães J, Ascensão A, Seabra AF, Rebelo AN (2013). Training status and match activity of professional soccer players throughout a season. Journal of Strength and Conditioning Research.

[ref-26] Someeh M, Norasteh AA, Daneshmandi H, Asadi A (2015). Influence of mulligan ankle taping on functional performance tests in healthy athletes and athletes with chronic ankle instability. International Journal of Athletic Therapy and Training.

[ref-27] Spink MJ, Fotoohabadi MR, Menz HB (2010). Foot and ankle strength assessment using handheld dynamometry: reliability and age-related differences. Gerontologia.

[ref-28] Tanen L, Docherty CL, Van Der Pol B, Simon J, Schrader J (2014). Prevalence of chronic ankle instability in high school and division I athletes. Foot and Ankle Specialist.

[ref-29] Wang H, Yu H, Kim YH (2021). Comparison of the effect of resistance and balance training on isokinetic eversion strength, dynamic balance, hop test, and ankle score in ankle sprain. Life.

[ref-30] Wikstrom EA, Tillman MD, Chmielewski TL, Cauraugh JH, Naugle KE, Borsa PA (2012). Discriminating between copers and people with chronic ankle instability. Journal of Athletic Training.

[ref-31] Wisthoff B, Matheny S, Struminger A, Gustavsen G, Glutting J, Swanik C, Kaminski TW (2019). Ankle strength deficits in a cohort of college athletes with chronic ankle instability. Journal of Sport Rehabilitation.

